# Bridging Cognitive Architecture and Developmental Measurement for Artificial General Intelligence

**DOI:** 10.3390/jintelligence14080179

**Published:** 2026-08-05

**Authors:** Yuqiu Fu, Yuxi Wang, Hongzhao Xie, Shiyun Zhao, Mingyuan Liu, Yujie Lu, Xinyi He, Zhenku Cheng, Yujia Peng, Zhenliang Zhang

**Affiliations:** 1 School of Psychological and Cognitive Sciences and Beijing Key Laboratory of Behavior and Mental Health, Peking University, Beijing 100871, China; 2 State Key Laboratory of General Artificial Intelligence, Beijing Institute for General Artificial Intelligence (BIGAI), Beijing 100080, China; 3 Institute for Artificial Intelligence, Peking University, Beijing 100871, China; 4 School of Intelligence Science and Technology, Peking University, Beijing 100871, China

**Keywords:** human intelligence, artificial general intelligence, intelligence assessment, cognitive architecture, intellectual development

## Abstract

Current artificial intelligence evaluation often relies on static benchmarks and task-specific performance metrics, which are useful for system comparison but limited in assessing developmental progression, cognitive architecture, adaptive transfer, and real-world applicability. Building on developmental approaches to AGI testing, including our earlier conceptual proposal, this article develops an operational formulation of the General–Specialized–Applicable (GSA) framework for artificial general intelligence (AGI) evaluation. The framework organizes evidence into three non-interchangeable stages: the General stage, which assesses foundational capacities for open-ended generalization, value-oriented regulation, and autonomy; the Specialized stage, which evaluates stable domain-specific competence; and the Applicable stage, which examines whether such competence can be deployed safely and robustly in realistic environments. The article further introduces a dynamic task-generation pipeline and a provisional operational rubric for interpreting stage-specific evidence. An illustrative case study using household tasks in a simulated embodied environment illustrates how GSA can provide diagnostic information beyond aggregate benchmark scores by identifying capability gaps in current multimodal large language model (MLLM) agents. Rather than offering a final universal standard, the GSA framework provides an evaluation-oriented structure for connecting benchmark performance with architectural readiness, specialization, and deployment-level applicability.

## 1. Introduction

The rapid development of artificial intelligence (AI), especially large-scale foundation models and autonomous agents, has renewed a central question in intelligence research: how should intelligence be conceptualized, measured, modeled, and evaluated? This question is important not only for AI research, but also for the broader study of human intelligence. Intelligence evaluation should not merely ask whether a system performs well on particular tasks. It should also examine what kinds of capacities underlie that performance, how those capacities are organized, how they develop, and whether they support adaptive action across changing real-world contexts.

Human intelligence research provides a useful starting point for addressing this issue. Since the emergence of modern intelligence testing, intelligence has rarely been treated simply as success on isolated tasks. Early intelligence tests, such as the Binet–Simon scale, were designed to identify children who needed educational support and already treated intelligence as a set of developing cognitive functions rather than as performance on a single problem (Binet & Simon, 1904). Spearman’s theory of general intelligence introduced the idea that performance across diverse mental tasks reflects both a general factor and task-specific abilities (Spearman, 1904). Later psychometric work further elaborated this structure through standardized intelligence scales and hierarchical models of cognitive abilities, including the Wechsler scales and the Cattell–Horn–Carroll framework, which distinguish general ability, broad cognitive domains, and narrower skills (Carroll, 1993; McGrew, 2005; Wechsler, 1939). At the same time, developmental and cognitive traditions emphasize that intelligent behavior changes over time and depends on the coordinated organization of perception, memory, language, reasoning, social understanding, and action planning. From this perspective, intelligence assessment should evaluate not only observable performance, but also the structure, developmental trajectory, transferability, and practical relevance of intelligent capacities.

AI evaluation has historically followed a different trajectory. Classic behavioral paradigms, such as the Turing Test, assessed machine intelligence through observable behavior and human-like responses (Copeland, 2000). Subsequent tests targeted more specific capacities, including commonsense reasoning in the Winograd Schema Challenge, creativity in the Lovelace Test, and constrained or human-like responses in the Minimum Intelligent Signal Test and the Loebner Prize competition (Bringsjord et al., 2001; Levesque et al., 2012; Łupkowski & Jurowska, 2019; Shah & Warwick, 2009). The aforementioned approaches played an important role in establishing behavioral criteria for machine intelligence. However, they mainly ask whether a system can produce appropriate outputs under specified conditions, offering limited insight into the internal organization, developmental structure, or adaptive transfer of intelligent capacities.

Recent AI evaluation has increasingly shifted toward large-scale benchmark-based assessment. Benchmarks such as the General Language Understanding Evaluation (GLUE) (A. Wang et al., 2018), Super General Language Understanding Evaluation (SuperGLUE) (A. Wang et al., 2019), Massive Multitask Language Understanding (MMLU) (Hendrycks et al., 2021a), MATH (Hendrycks et al., 2021b), Multi-modal Benchmark (MMBench) (Liu et al., 2024), MathVista (Lu et al., 2024), Beyond the Imitation Game Benchmark (BIG-bench) (Srivastava et al., 2023), HellaSwag (Zellers et al., 2019), and TruthfulQA (Lin et al., 2022) have made it possible to quantify performance, compare systems, and document progress across language understanding, knowledge reasoning, mathematical problem solving, multimodal processing, and truthfulness. To improve source verifiability, this overview prioritizes peer-reviewed conference or journal versions of benchmark studies and omits preliminary preprints where suitable published alternatives are available. These benchmarks are valuable, but they remain limited as measures of intelligence in a broader sense. Most focus on observable outcomes, such as accuracy, completion rate, or task-specific utility. They provide less information about how capacities emerge, interact, generalize, or support situated and socially meaningful action.

This limitation becomes especially clear when evaluating AGI. Fixed benchmarks are built on predefined item distributions and task conditions. As a result, they are less suited to assessing capacities such as cross-context generalization, grounded interaction, and embodied adaptation when tasks extend beyond predefined item distributions (Brooks, 1991; Harnad, 1990; B. Lake & Baroni, 2018; Moskvichev et al., 2023). This does not mean that benchmarks are unimportant. Rather, benchmark scores should be understood as one form of evidence within a broader framework for evaluating the structure, development, and application of intelligence.

Several recent approaches have attempted to broaden AI evaluation beyond single-task performance. Early discussions of AI ability levels, DeepMind’s AGI levels, and the Holistic Evaluation of Language Models (HELM) methodology provide useful taxonomies and evaluation dimensions (Kak, 1996; Liang et al., 2023; Morris et al., 2024). These approaches expand the scope of AI assessment. Yet many still emphasize endpoint performance, ability classification, or broad evaluative checklists. They provide less guidance on how different forms of evidence relate to the structure and development of intelligence.

Taken together, existing evaluation paradigms have limitations that yield insufficient evidence regarding the cognitive architecture, developmental organization, and adaptive applicability of intelligent capacities. Behavioral tests assess whether a system can generate appropriate responses, yet they do not explain the cognitive structures or processes underlying those behaviors. Task-oriented benchmarks quantify performance under predefined conditions, but their scope is often constrained by fixed task distributions and evaluation settings. Broader evaluation frameworks expand the range of criteria, but they remain only loosely connected to theories of cognitive architecture, developmental progression, and real-world adaptation. General intelligence, however, is usually understood as more than success across many tasks; it involves flexible generalization, abstraction, autonomous adaptation, and value-sensitive regulation (Goertzel & Pennachin, 2007; B. M. Lake et al., 2017). AGI evaluation therefore requires a framework that connects task performance with the organization, development, transfer, and real-world applicability of intelligent capacities.

A recent human-intelligence-inspired account has already outlined a developmental route for AGI evaluation from general abilities to specialized abilities and application (Peng et al., 2026). That account served as an initial conceptual proposal, but it mainly introduced the idea of a developmental testing trajectory and a limited set of child-referenced embodied examples. It did not attempt to specify, in a systematic way, what forms of evidence should be evaluated at each stage, how developmental measurement should be connected to cognitive architecture, nor did it provide large-scale model-agent experiments or specify how such results should be interpreted as staged evaluation evidence.

To address this need, the present article develops the General–Specialized–Applicable (GSA) framework as a more fully specified methodology for AGI evaluation. Drawing on human intelligence research, including psychometrics, formal models of ability, intellectual development, cognitive architecture, and applied assessment methods, the GSA framework treats evidence of intelligence as developmental and stage-specific. GSA distinguishes among general capacities, specialized competence, and real-world applicability, which are related but not interchangeable forms of evidence. A high score on a fixed task set may indicate strong domain-specific competence, but it does not by itself establish general intelligence or robust applicability in realistic environments.

The contribution of the present article is therefore not the first proposal that AGI evaluation can be developmental. Rather, it extends a preliminary developmental proposal into a comprehensive account of GSA as an evaluation framework: it links developmental measurement with cognitive-architecture-centered assessment, specifies stage-specific evidential requirements, introduces a dynamic task-generation and scoring procedure, and illustrates how staged interpretation can diagnose capability gaps in current model agents. Benchmark results remain important, but they should be interpreted alongside evidence of generalization, autonomy, value-sensitive regulation, robustness, and context-sensitive adaptation.

By linking AGI evaluation to broader questions in intelligence research, the GSA framework provides a theoretical structure for assessing the organization, development, and application of intelligent capacities in artificial systems. The scope of the present article is deliberately focused. Rather than reviewing every tradition of intelligence assessment or presenting a universally validated standard, it develops a stage-specific evaluation framework and illustrates how this framework can organize evidence from current AI systems. Below, the article defines the operational meaning of the three GSA levels, describes a dynamic task-generation and evaluation procedure, and illustrates the framework through developmentally inspired embodied tasks in simulated household environments.

## 2. The General–Specialized–Applicable Framework

### 2.1. Design Principles of the GSA Evaluation Framework

The GSA framework provides a way to organize the evaluation of interdependent architectural capacities that are relevant to general, adaptive, and value-aligned intelligence. The formulation below builds on the previously proposed conceptual GSA developmental trajectory (Peng et al., 2026) and refines it into an evaluation framework centered on cognitive architecture, stage-specific measurement, and operational evidence.

#### 2.1.1. Cognitive-Architecture-Centered Evaluation

To operationalize an architecture-centered view of AGI evaluation, the General stage must specify which foundational capacities should be examined before specialized competence or real-world applicability is interpreted. The CUV framework of He et al. (2025) provides the theoretical basis for this content. CUV identifies three interdependent components of AGI: learning potential functions (U), value functions (V), and cognitive architecture (C), and emphasizes that general intelligence cannot be inferred from task performance alone.

In GSA, these CUV components are translated into three evaluation dimensions: F1 open-ended task generalization, F2 value-oriented regulation, and F3 cognitive architecture-enabled autonomy. The seven G-stage aspects further elaborate these dimensions for assessment: physical intelligence and social intelligence under F1; value acquisition and value-driven behavior under F2; and explainability, security and trust behavior, and self-awareness under F3. Thus, the G-stage structure retains a direct conceptual relationship with CUV while converting its components into an evaluation-oriented taxonomy.

The additional role of GSA is to embed this CUV-derived G-stage structure within a developmental measurement pathway. The Specialized stage evaluates whether foundational capacities consolidate into stable domain-specific competence, and the Applicable stage evaluates whether general and specialized capacities remain effective under realistic deployment constraints. GSA therefore specifies how CUV-informed capacities can be measured, interpreted, and connected across General, Specialized, and Applicable stages.

**U-Task generalization:** the ability to successfully accomplish unseen tasks and challenges within the trained knowledge space.

**V-Value system:** possessing an internal value structure that guides decision-making and behavior.

**C-Cognitive architecture-enabled autonomy:** the ability to autonomously generate goals and task ideas based on intrinsic value orientations and contextual assessment.

Together, these operationalized capacities form an interdependent mechanism in which value alignment shapes goal formation, cognitive architecture enables self-directed decision-making and autonomous task generation, and generalization ensures that such decisions can be successfully realized in previously unseen scenarios.

In this sense, value-driven regulation enables the agent to interpret its interaction context and generate contextually appropriate goals, while generalization supports the execution of these self-generated goals in novel but structurally related situations, without requiring task-specific retraining.

#### 2.1.2. Developmental Evaluation Paradigm

Beyond its emphasis on cognitive-architecture-centered evaluation, the GSA framework is distinguished by a second defining characteristic: its explicitly developmental nature. Here, the term “developmental” refers to the staged organization and growth-oriented design of the evaluation framework itself.

Unlike age-stratified single-task assessments such as intelligence quotient (IQ)-style tests, for example, the Wechsler Intelligence Scales or Stanford–Binet tests, which reflect development primarily through norm-referenced score differences across age groups, the proposed developmental paradigm evaluates AGI along a progressive trajectory grounded in its underlying cognitive architecture. Specifically, GSA begins with the assessment of general cognitive and value-related capacities, namely the General stage, extends to the evaluation of domain-specific skill acquisition, namely the Specialized stage, and ultimately examines performance in real-world or application-oriented settings, namely the Applicable stage.

Importantly, the developmental property of GSA is not limited to stage ordering. In the Specialized and Applicable stages, the framework does not rely on a fixed or closed set of evaluation tasks; instead, it is designed to continuously accommodate newly introduced tasks and scenarios. This open-ended structure ensures that development is assessed not as optimization over static benchmarks but as an agent’s sustained capacity for growth, adaptation, and transfer over time.

#### 2.1.3. Real-World-Oriented Application Evaluation

While benchmark-based evaluations verify domain-specific knowledge and skills, they primarily assess performance under controlled and predefined conditions. High benchmark scores may therefore need to be complemented by additional evidence concerning transfer, robustness, and sustained performance in dynamic real-world environments.

Building upon this foundation, the GSA framework situates benchmark-driven testing within a broader evaluation structure that also includes real-world-oriented validation. Application competence is therefore conceptualized as requiring validation in realistic interaction contexts, rather than being inferred solely from closed examination settings. This shift aims to assess whether acquired skills can be robustly transferred, adapted, and executed under real-world conditions, as illustrated by the application-oriented examples in Table 1.

In the following sections, we detail the concrete evaluation methodologies corresponding to the three stages of the GSA framework—General, Specialized, and Applicable—highlighting how each stage incrementally advances from architectural foundations to domain specialization and finally to real-world validation.

### 2.2. G-Stage: General Stage Evaluation

**Figure 1:**
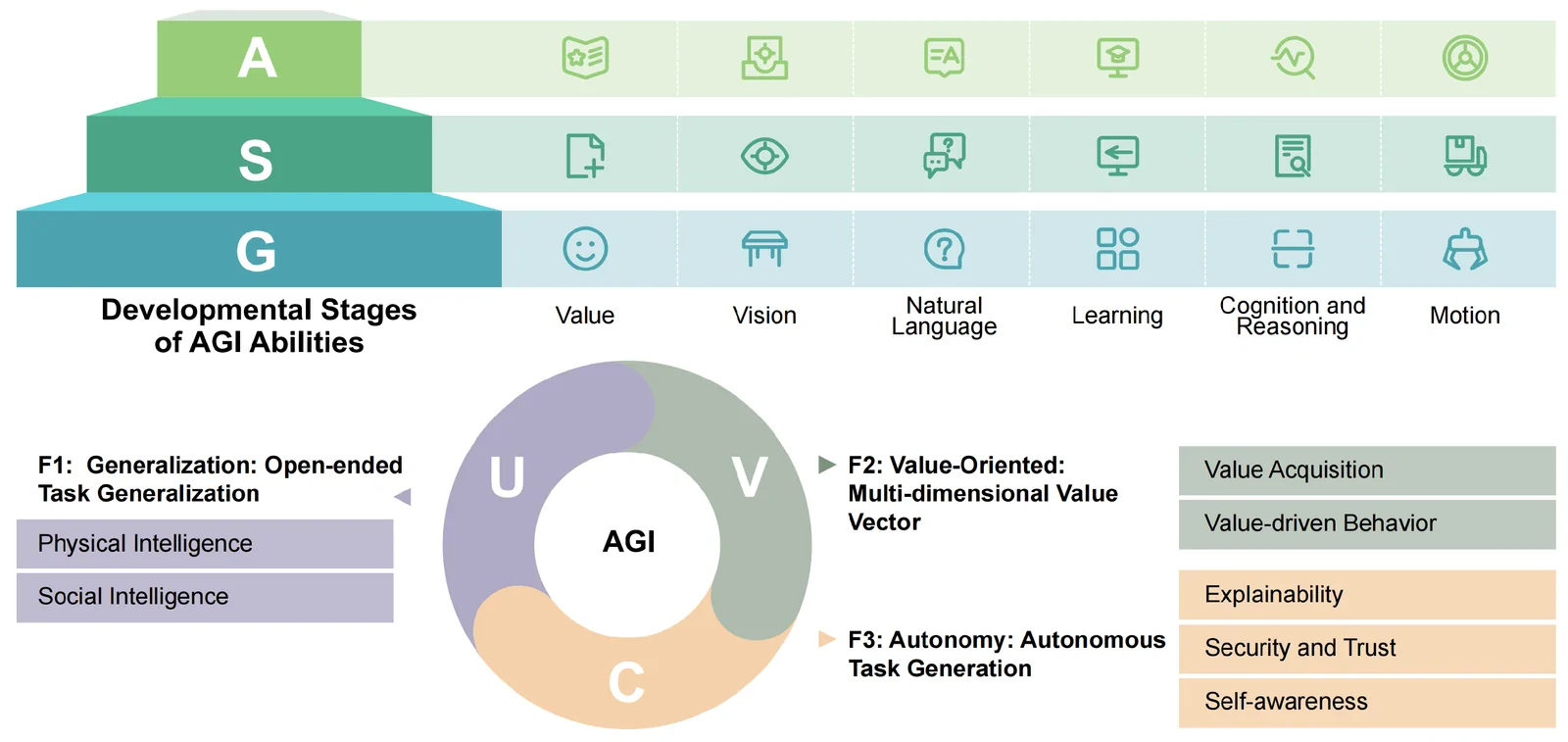
Overview of the GSA evaluation framework. The General (G) stage assesses foundational cognitive–architectural capacities through an evaluation-oriented operationalization of the CUV components: open-ended task generalization (U), value-oriented regulation (V), and cognitive architecture-enabled autonomy (C), together with seven associated G-stage aspects. The Specialized (S) stage evaluates the consolidation of domain-specific competencies built upon G-stage general capacities under structured task settings. The Applicable (A) stage examines whether general and specialized abilities can be deployed in real-world or realistic operational environments under practical constraints.

The General (G) stage evaluates whether an agent possesses the foundational cognitive–architectural capacities required for open-ended generalization, value-oriented regulation, and autonomous behavior. Rather than assessing task-specific proficiency, this stage focuses on whether the agent exhibits stable internal mechanisms that support continual ability development across environments. As illustrated in the GSA framework (Figure 1), the General stage is organized around three core architectural mechanisms—generalization (U), value orientation (V), and cognitive architecture-enabled autonomy (C). These mechanisms are operationalized through seven complementary evaluation aspects, which together define the concrete testing scope of the G stage.

#### 2.2.1. F1: Generalization—Open-Ended Task Generalization

The first mechanism, generalization, evaluates whether an agent can transfer learned representations and strategies to previously unseen tasks, environments, and interaction settings. In the G stage, this capacity is operationalized as open-ended task generalization, namely the ability to engage with tasks sampled from an explicitly defined and expandable generative space rather than from a fixed benchmark list. This terminology is intended to avoid the implication that an agent can be tested on literally infinite tasks. The diagnostic question is whether performance remains stable across newly generated but structurally coherent task variants, such that success cannot be explained by memorization of a closed task distribution. The evaluation of open-ended task generalization is detailed in Section 4; here, we describe the two aspects through which it is assessed at the G stage.

Table 1 is intended as an illustrative stage mapping rather than an exhaustive taxonomy. Across the six dimensions, G-stage examples refer to foundational architectural capacities that can support further learning and transfer, S-stage examples refer to stable domain-specific competence under structured task conditions, and A-stage examples refer to the deployment of those capacities in realistic workflows where robustness, safety, and social consequences matter. Thus, a complex semantic-reasoning task is placed at the S stage when it reflects consolidated language-domain competence, whereas the G stage concerns the more general capacity to form and transfer representations that can later support such competence.

Physical intelligence examines whether agents can generalize across physical or simulated environments that require the integration of perception, reasoning, and action under continual novelty. Representative evaluations include open-ended task generation and randomized task curricula, where agents are exposed to dynamically varying tasks rather than fixed benchmarks. For example, Active Task Randomization assesses whether agents can acquire robust skills through unsupervised exposure to diverse and feasible tasks, providing a concrete test of generalization beyond predefined task distributions (Fang et al., 2023). Recent embodied-agent benchmarks and generative-simulation pipelines further extend this paradigm. Planning And Reasoning Tasks in humaN–Robot collaboration (PARTNR) provides large-scale collaborative household tasks, whereas RoboGen uses generative simulation to produce diverse robotic training and evaluation experiences (Chang et al., 2025; Y. Wang et al., 2024).

Social intelligence evaluates generalization in interactive and multi-agent contexts, where agents are expected to infer intentions, adapt communication strategies, and coordinate behavior under partial observability. Such settings test whether generalization extends beyond physical variation to social and communicative dynamics. Embodied benchmarks such as PARTNR examine planning and coordination in collaborative household tasks, whereas assistant-oriented benchmarks such as GAIA assess reasoning, multimodal information integration, and tool use across heterogeneous real-world questions (Chang et al., 2025; Mialon et al., 2024).

#### 2.2.2. F2: Value-Oriented Behavior—Multi-Dimensional Value Vector

The second mechanism, value orientation, examines whether an agent’s behavior is regulated by stable internal value representations rather than purely by immediate rewards. At the G stage, value orientation is assessed through two closely related aspects: value acquisition and value-driven behavior.

Value acquisition evaluates whether agents can form, update, and maintain multiple value dimensions such as safety, reliability, or preference consistency, through interaction with their environment. This aspect focuses on whether values emerge as persistent internal representations rather than externally imposed constraints.

Value-driven behavior examines whether learned values meaningfully constrain planning and action selection across contexts. Although existing calibration, selective-prediction, and truthfulness evaluations do not directly measure value acquisition, they can provide limited behavioral evidence concerning uncertainty management, unsupported responding, and conservative action selection (Geifman & El-Yaniv, 2019; Guo et al., 2017; Lin et al., 2022). Multi-dimensional agent benchmarks such as GAIA provide partial tests of reasoning, tool use, and robustness across diverse assistant-oriented scenarios, but do not directly assess stable value-oriented regulation (Mialon et al., 2024).

#### 2.2.3. F3: Autonomy—Autonomous Task Generation

The third mechanism, autonomy, evaluates whether an agent can independently generate goals, tasks, and action sequences without explicit external instruction. In the G stage, autonomy is operationalized through three evaluation aspects: self-awareness, explainability, and security/trust behavior.

Self-awareness assesses whether agents can monitor internal states, recognize uncertainty, and identify their own epistemic boundaries. This includes the ability to judge when additional information or external assistance is required. Behavioral proxies relevant to this aspect include calibration, selective-prediction, and truthfulness evaluations, which examine whether systems can estimate uncertainty, withhold unreliable answers, or adjust responses when evidence is insufficient (Geifman & El-Yaniv, 2019; Guo et al., 2017; Lin et al., 2022).

Explainability evaluates whether agents can provide coherent and faithful explanations for their decisions, plans, and task generation processes. It is treated as a component of autonomy, as autonomous agents should be able to account for self-generated goals and actions in a manner that can be inspected and regulated by humans.

Security and trust evaluate whether autonomy is exercised within safety and trust boundaries. This includes avoiding unsafe actions, resisting opportunistic reward maximization, and maintaining consistency with internal value constraints.

As shown in Figure 1, the General (G) stage is organized around three core functional dimensions (F1–F3), from which seven concrete evaluation aspects are derived. Together, these aspects operationalize the G stage as an architecture-centered assessment of AGI readiness. Passing the G stage would provide evidence that generalization, value orientation, and autonomy are sufficiently integrated for the purposes of G-stage evaluation, providing a stable foundation for subsequent specialization and real-world application in the S and A stages of the GSA framework.

The present section describes the evaluative content of the G stage—what capacities are assessed and through which aspects. The criteria by which a system is judged to have satisfied G-stage requirements, including operationalized scoring procedures and passing thresholds, are detailed in Section 3.

### 2.3. S-Stage: Specialized Stage Evaluation

The Specialized (S) stage evaluates whether an agent can reliably extend its general cognitive architecture into constrained domains that require structured reasoning and domain-specific competence. Unlike the General stage, which focuses on architectural readiness, the S stage assesses the consolidation and stability of specialized competencies under controlled task settings.

At this stage, performance-based metrics regain interpretive relevance; however, within the GSA framework, such metrics are treated as indicators of architectural extension rather than standalone measures of intelligence. Specialized evaluation examines whether domain-specific skills emerge through principled adaptation of the agent’s general mechanisms rather than through brittle memorization or heuristic shortcuts.

S-stage evaluations are designed around well-defined task families that require hierarchical reasoning, abstraction, and strategy refinement. Typical evaluation settings include structured problem sets, adversarial perturbations, and domain-shift scenarios, which test whether learned strategies remain stable under variation while preserving value consistency and safety constraints. These tests probe representational depth, compositional reasoning, and the consolidation of domain knowledge.

Specialized evaluation at this stage can be instantiated through domain-focused planning and reasoning benchmarks. For example, PARTNR evaluates structured planning and reasoning in embodied multi-agent environments (Chang et al., 2025), while GAIA evaluates general assistants on real-world questions requiring reasoning, multimodality, web browsing, and tool use (Mialon et al., 2024).

Importantly, the S stage does not require holistic intelligence across domains. Instead, it assesses whether the agent can achieve reliable, transferable competence within specific domains, thereby establishing a stable bridge between general architectural capacity and real-world deployment. As with the G stage, the present section describes the scope and content of the S-stage evaluation. Operational criteria for S-stage assignment, including success rate thresholds and robustness requirements, are specified in Section 3.

### 2.4. A-Stage: Applicable Stage Evaluation

The Applicable (A) stage evaluates whether an agent can robustly deploy its general and specialized abilities in real-world or realistic operational environments under practical constraints. This stage shifts evaluation from controlled task performance to sustained interaction under deployment conditions, including safety, robustness, reliability, and social alignment. Importantly, the A stage is better understood in terms of deployment conditions, failure costs, and long-horizon interaction dynamics, rather than as a simple extension of benchmark difficulty.

A-stage evaluation examines how agents operate in human-centered contexts with underspecified objectives, dynamic environments, and real consequences of failure. Rather than isolated task success, it emphasizes behavioral stability, adaptation to unforeseen conditions, and sustained alignment with value and safety requirements, including whether failures remain recoverable or lead to unsafe, irreversible outcomes.

Application-oriented evaluation is illustrated by benchmarks that emphasize realistic or physically grounded deployment. For example, RoboGen assesses whether agents can acquire large-scale experience through generative simulation for real-world robotic learning (Y. Wang et al., 2024), while published agent and assistant benchmarks probe service reliability, tool use, and robustness in open-ended interaction settings (Liang et al., 2023; Mialon et al., 2024).

To operationalize application-level evaluation, the A stage adopts a dual-track framework spanning virtual service environments and physically embodied real-world settings. As summarized in Table 2, evaluation extends beyond correctness on predefined tasks to assess whether general and specialized abilities yield dependable performance under deployment constraints. By incorporating failure costs, safety considerations, and long-horizon dynamics, the A stage serves as the final validation step of the GSA framework, determining whether architectural coherence and specialized competence translate into intelligence that is viable in practice. The present section describes the conceptual scope of A-stage evaluation. Quantitative passing criteria and deployment duration requirements are specified in Section 3.

### 2.5. Comparison with Other Evaluation Frameworks

As summarized in Table 3, existing evaluation frameworks most closely align with the S-stage in GSA, providing limited operationalization of cross-context generalization, autonomous task generation, or deployment-level robustness. The GSA framework extends this paradigm by organizing evaluation across three complementary stages. The G-stage targets foundational cognitive capacities that reflect architectural properties rather than task-specific skills. The Specialized (S) stage consolidates domain competence within structured task families. The Applicable (A) stage evaluates whether these abilities remain robust, safe, and aligned under real-world deployment conditions.

Together, these stages span the full spectrum from architectural readiness to practical applicability. This staged design broadens the interpretation of AGI evaluation by linking benchmark performance with developmental and architectural properties. Rather than interpreting performance as a snapshot of competence, GSA emphasizes an agent’s developmental potential, robustness across contexts, and ability to transfer skills under changing conditions, as summarized in Table 3.

Each stage plays a distinct and non-overlapping role. The G stage captures the architectural foundations of general intelligence. The S stage unifies existing task benchmarks within a coherent specialization layer. The A stage introduces a human-centered perspective by testing long-horizon interaction, safety, and value alignment in realistic environments.

In this comparison, the main distinction is not that GSA introduces more difficult tasks, but that it assigns different evidential roles to different forms of evaluation. Conventional benchmarks are most informative about bounded task competence, whereas the GSA framework separates evidence for architectural readiness, specialized competence, and deployment-level applicability. The next section translates this distinction into an operational rubric for assigning systems to the G, S, and A stages.

### 2.6. Relationship to Existing AGI and Cognitive-Architecture Literature

Existing AGI evaluation and cognitive-architecture studies address several related but distinct questions: how systems generalize, how capacities are acquired, how cognitive processes are organized, and how broadly performance is demonstrated. GSA uses these distinctions to clarify the evidential role of each research tradition. In the narrower line of developmental AGI testing, the earlier GSA-oriented proposal identified a human-developmental route from general ability to specialization and application, but left open how stage claims should be measured and interpreted (Peng et al., 2026). The present formulation builds on that conceptual route by specifying an evidential structure that connects developmental testing with cognitive-architecture-centered evaluation.

Within this landscape, abstraction-and-reasoning benchmarks provide a concrete example of evidence relevant to F1. The Abstraction and Reasoning Corpus (ARC) literature examines abstraction and sample-efficient generalization through theoretical accounts, concept-focused benchmarks, computational approaches, and human behavioral datasets (Bober-Irizar & Banerjee, 2024; Chollet, 2019; LeGris et al., 2025; Moskvichev et al., 2023). GSA shares this generalization-centered concern, but does not treat generalization alone as sufficient evidence of general intelligence. Within GSA, ARC-like results provide evidence about one G-stage dimension, while full G-stage evidence also requires value-oriented regulation and autonomy, and higher-stage claims require specialized stability and deployment-level robustness.

Developmental robotics addresses how intelligent capacities can be acquired through embodied interaction, sensorimotor learning, and progressive adaptation (Cangelosi & Schlesinger, 2015). This process-oriented tradition is relevant to GSA because it emphasizes that competence develops through continuing interaction rather than appearing only as an endpoint score.

Classical cognitive architectures address the related but distinct question of how capacities are organized and coordinated within an agent. Soar, ACT-R, and LIDA model the integration of perception, memory, learning, decision-making, and action selection (Anderson, 2007; Franklin et al., 2014; Laird, 2012). GSA is not itself a cognitive architecture; its evaluative role is to specify what evidence would justify claims that such organized capacities are integrated, transferable, and usable beyond isolated task success.

Whereas the preceding traditions focus on the acquisition and organization of cognitive capacities, recent evaluations of frontier models and general assistants focus on the breadth of demonstrated task performance. These studies provide broader evidence of cross-domain competence and, in some cases, partial transfer, but they do not by themselves establish the complete GSA evidence profile. Capability studies such as Sparks of Artificial General Intelligence and the GPT-4 technical report examine performance across many domains (Bubeck et al., 2023; OpenAI, 2023), while GAIA and MathVista probe assistant-style tool use, multimodal reasoning, and visual mathematical problem solving (Lu et al., 2024; Mialon et al., 2024). In GSA terms, these studies are most directly informative about specialized competence and partial G-stage generalization. As work on shortcut learning cautions, however, high performance can still reflect brittle heuristics rather than robust transfer (Geirhos et al., 2020). GSA therefore treats benchmark and case-study performance as one source of staged evidence, to be complemented by independent evidence concerning value-oriented regulation, autonomy, and deployment readiness.

## 3. Operational Rubric for G, S, and A Stage Assignment

The GSA framework specifies three levels of intelligence evidence. To make stage assignment interpretable and reproducible, this section translates each stage into an operational rubric that specifies what evidence is required, what it demonstrates, and what it does not. A central premise of the rubric is that the three stages are distinguished by the type of evidence required, not simply by task difficulty. A harder benchmark does not automatically imply a higher stage; each stage asks a qualitatively different question about the system’s capacities. Importantly, the operational rubric proposed here represents one feasible implementation of the GSA framework rather than a definitive scoring standard. Its formulas, parameters, and thresholds are intended to make stage assignment explicit and auditable, and should be further calibrated through empirical testing across diverse systems and domains.

To handle partial and domain-specific evidence, stage assignment should be reported as a domain-qualified profile rather than as a simple global label. We propose three provisional rules. First, the rules are non-compensatory: strong evidence on one core dimension cannot compensate for missing evidence on another required dimension. For example, high benchmark performance cannot replace evidence of value-oriented regulation or autonomy. Second, stage claims should be domain-qualified, such as “partial G-stage evidence in simulated household tasks,” rather than generalized to AGI as a whole. Third, reports should distinguish full evidence, partial evidence, and insufficient evidence. A full stage claim requires that all required dimensions for that stage meet the specified minimum criteria; partial evidence indicates that some but not all dimensions are satisfied; insufficient evidence indicates that one or more required dimensions were not measured. Operationally, the relevant dimensions should follow a separate-tests, joint-judgment logic: generalization, value-oriented regulation, autonomy, robustness, and deployment reliability should be assessed separately for diagnostic clarity, while the final stage interpretation should be made jointly according to whether the required evidence profile is complete. This profiling approach preserves the staged logic of GSA while avoiding overconfident binary classification.

### 3.1. G-Stage

The G-stage assignment requires evidence that the system possesses foundational generalization, value-oriented regulation, and bounded autonomy as integrated architectural properties rather than as task-specific skills. Three kinds of evidence are specifically required: task generalization, value-oriented regulation, and autonomous task generation.

First, task generalization evaluates performance across both seen and unseen task families under identical conditions, without additional fine-tuning. Task families are defined as sets of tasks sharing an underlying structural logic while varying in surface form—objects, layouts, contexts, or environmental conditions. The key criterion is not whether the system has encountered a specific task template, but whether its competence transfers across variations that preserve task structure while changing surface realization. To operationalize generalization, it can be scored as:

(1) T=μ−γ·σ

where *T* denotes the task generalization score, μ denotes mean performance across task categories, σ denotes the standard deviation of performance across task categories, and γ controls the penalty for performance variability across task categories. This formulation rewards systems that are both accurate on average and stable across categories, rather than systems that perform well in some task families but fail in others. Consistent with the household-environment testing specification, γ may be set in the range of 0.5–2.0 in general testing scenarios, with larger values imposing a stronger penalty on cross-category instability.

Second, value-oriented regulation evaluates whether the system’s behavior is constrained by stable value and safety considerations rather than by task completion alone. In G-stage evaluation, this component assesses whether the system can avoid unsafe, irrelevant, deceptive, or socially inappropriate actions, especially when task goals are underspecified or when multiple plausible actions are available. In household or human-centered environments, value-oriented regulation may be evaluated through indicators such as safety compliance, appropriateness of action selection, avoidance of harmful or unnecessary behavior, consistency with explicit user preferences, and alignment with legal, ethical, and social norms. Because the operational measurement of value systems remains less mature than performance-based task metrics, the present framework treats value-oriented regulation as a required qualitative and quantitative evidence dimension. Its scoring procedure should be explicitly reported according to the evaluation context, rather than being inferred from task success alone.

As a provisional implementation in a household simulation, value-oriented regulation can be scored at the level of value-relevant decisions. A value-relevant decision is any action or refusal that may affect safety, social appropriateness, user preference, unnecessary harm, or uncertainty management. Each decision can be coded as 0 = violation, 1 = partially acceptable, or 2 = value-consistent by trained raters using a predefined rubric. A complementary binary indicator can record whether any critical violation occurred in a task. One simple summary score is the Value Consistency Rate:

(2) $$VCR=\frac{N_{\mathrm{consistent}}}{N_{\mathrm{relevant}}}$$

where VCR denotes the Value Consistency Rate, $N_{\mathrm{consistent}}$ is the number of value-consistent decisions, and $N_{\mathrm{relevant}}$ is the total number of value-relevant decisions. Value-consistent decisions include those rated as appropriate with respect to safety, social norms, explicit user preferences, avoidance of unnecessary or harmful actions, and conservative behavior under uncertainty. This protocol remains preliminary, but it makes the V dimension reproducible enough to be reported alongside task success and autonomy indicators.

Third, autonomous task generation requires the system to generate tasks on its own rather than only execute externally assigned ones. Generated tasks are assessed along two dimensions—task relevance (TR), which measures whether they align with genuine task-domain needs, and task coverage (TC), which measures whether they span a sufficiently diverse range of task categories. Hence, the two terms can be combined as:

(3) ATG=α·TR+(1−α)·TC

where ATG denotes the autonomous task generation score, TR denotes task relevance, TC denotes task coverage, and α controls the relative weight assigned to relevance. Following the local testing specification, α may be set to 0.5 in general testing scenarios to balance task relevance and task coverage. Test designers may also adjust α according to specific evaluation purposes, such as placing greater emphasis on practical relevance, diversity, innovation, or safety, provided that the chosen value and its rationale are explicitly reported.

Passing the G stage means the system demonstrates foundational cross-task transfer and meaningful autonomous task generation. It does not establish reliable domain competence or deployment readiness. A system that performs well on a structured benchmark through narrow specialization, without showing robust generalization across task families, does not satisfy G-stage requirements regardless of its aggregate benchmark score.

### 3.2. S-Stage

S-stage evaluation requires evidence that G-stage capacities have been consolidated into stable, transferable competence within specific task domains. Evaluation at this stage uses curated task portfolios with explicit success criteria under controlled conditions. The central criteria are repeatability, robustness under surface variation, and resistance to adversarial perturbation.

For example, language understanding benchmarks such as GLUE (A. Wang et al., 2018) and SuperGLUE (A. Wang et al., 2019), knowledge and reasoning benchmarks such as MMLU (Hendrycks et al., 2021a), mathematical reasoning benchmarks such as MATH (Hendrycks et al., 2021b), multimodal benchmarks such as MMBench (Liu et al., 2024) and MathVista (Lu et al., 2024), and broader diagnostic suites such as BIG-bench (Srivastava et al., 2023), HellaSwag (Zellers et al., 2019), and TruthfulQA (Lin et al., 2022) can be used as candidate S-stage instruments. Within GSA, however, these benchmarks should not be treated as direct evidence of general intelligence. Instead, they provide evidence that an agent has acquired reliable competence within particular task families or knowledge domains. Their evidential value depends on whether performance is robust across task variations, whether competence transfers to related but unseen tasks, and whether the system can maintain performance without narrow benchmark-specific optimization.

Concretely, as an illustrative operational default rather than a universal cutoff, a system may be considered to have reached the S stage when it achieves at least 90% success across a specialized task portfolio, averaged over no fewer than 100 trials per task, with performance degradation across repeated sessions not exceeding 5%. These values are illustrative operational choices rather than thresholds derived directly from prior psychometric standards. The use of repeated trials and stability criteria is broadly informed by principles of binomial estimation, reliability, and score interpretation (Agresti & Coull, 1998; American Educational Research Association et al., 2014; Nunnally & Bernstein, 1994); the specific values should be further calibrated across systems, domains, and evaluation contexts.

Critically, performance at the S stage is interpreted as evidence of stable architectural extension rather than as a standalone measure of intelligence. A system may achieve high S-stage scores through heuristic memorization rather than principled generalization, and this distinction should be reflected in the diagnostic scoring procedure—specifically, through evaluation of whether competence holds under domain-shift scenarios and adversarial perturbations, not only under repeated exposure to the training distribution. Passing the S stage indicates that general capacity has been consolidated into domain competence; it does not establish readiness for real-world deployment.

### 3.3. A-Stage

A-stage assignment requires evidence that S-stage competence remains robust, safe, and recoverable under realistic deployment conditions. The evaluative question shifts from benchmark performance to situated validation.

Candidate A-stage implementations may include interactive household simulations, embodied navigation and manipulation environments, long-horizon planning tasks, tool-use platforms, human–agent collaboration settings, safety-critical decision scenarios, and domain-specific application environments in education, healthcare, scientific research, or administrative work. Here, evaluation should examine whether specialized capacities can be transferred, adapted, and sustained under realistic constraints, including environmental uncertainty, changing goals, incomplete information, social interaction, and value-sensitive trade-offs.

In the A stage, five properties are assessed concurrently (see Table 4 for one possible implementation). The threshold values in Table 4 should be understood as illustrative operational defaults rather than universal cutoffs.

In practice, they should be calibrated according to deployment risk, task domain, baseline human or expert performance, statistical uncertainty, and applicable safety or regulatory requirements. For example, lower-risk service applications may tolerate moderate failure and recovery thresholds, whereas safety-critical domains should require substantially stricter criteria, especially for critical safety incidents and value consistency. This risk-calibrated approach is consistent with AI risk management frameworks that emphasize context-specific measurement, monitoring, and mitigation of reliability, robustness, safety, and validity risks (ISO/IEC, 2023; National Institute of Standards and Technology, 2023), as well as psychometric principles that score interpretation should consider reliability, measurement error, and intended use (American Educational Research Association et al., 2014). As an illustrative default, deployment-level metrics may be monitored over one month of continuous operation, with weekly performance not falling below 80% of the initial level on any single indicator.

These requirements operationalize a distinction that benchmark scores cannot capture: the difference between a system that can complete tasks under controlled conditions and a system that remains dependable when conditions are uncertain, failures are costly, and human interaction is ongoing. The A stage is not defined as “more difficult tasks” but as evidence of deployment readiness under the full set of real-world constraints.

Stage assignment is cumulative but non-interchangeable. A system can be described as showing full G-stage evidence only if it demonstrates the required minimum evidence for open-ended task generalization, value-oriented regulation, and bounded autonomy in the specified domain. It can be described as showing S-stage evidence only if the relevant G-stage prerequisites are also satisfied and domain competence remains stable under controlled variation. It can be described as showing A-stage evidence only if G- and S-stage requirements are met and performance remains safe, recoverable, and robust under realistic deployment constraints. When some dimensions are missing or fail to meet the criterion, the appropriate conclusion is a partial evidence profile rather than a full stage label. This non-compensatory rule is essential: strong S-stage evidence does not replace G-stage evidence, and strong benchmark performance does not establish A-stage applicability. Stage assignment is therefore a matter of whether the available evidence supports the corresponding architectural and developmental claims within a specified domain, not a matter of crossing a single aggregate performance threshold.

## 4. Dynamic Task Generation Pipeline

The GSA framework requires that evaluation tasks not be treated as a fixed, closed benchmark list. Instead, tasks should be instances drawn from explicitly defined generative structures, so that the evaluation can expand as new domains and capacities become relevant. This section describes the dynamic task generation pipeline that underlies all three stages of the framework.

The core idea is that tasks are not treated as a fixed list of benchmark items but as instances drawn from explicitly defined generative structures. A task family specifies the cognitive construct to be evaluated—such as object-quantity understanding, spatial relational reasoning, or goal-directed social action—along with the environment, entity types, permissible action space, task constraints, success conditions, and controlled sources of variation. Once a task family is defined, individual task instances are produced by recombining its generative variables: object identity, quantity, spatial configuration, temporal order, goal condition, action requirement, social context, and value or safety constraint. Because the generative components are explicit and documented, the resulting task space can, in principle, be expanded in an open-ended but controlled manner, and the expansion is transparent and reproducible rather than arbitrary.

Task generation proceeds through four steps, described in turn below: (1) candidate generation, (2) filtering, (3) diversification, and (4) evolution.

First, candidate generation produces task instances by systematically varying the generative components within a defined family. For example, a household-task family might yield instances such as counting objects on a shelf, selecting a contextually appropriate gift, assembling blocks under spatial constraints, or organizing items in line with a social norm. The surface form of each instance differs, but all share the underlying task structure defined by the family. This is what makes cross-instance performance informative about generalization rather than memorization.

Second, filtering retains only candidates that satisfy four criteria. Feasibility requires that the task is completable within the environment and action space. Semantic validity requires that the task is contextually coherent and does not depend on undefined or nonsensical object relations. Structural consistency requires that tasks within the same family impose equivalent cognitive demands despite surface variation. Difficulty calibration requires that failures are diagnostic of the target capacity rather than caused by ambiguous instructions, missing affordances, or accidental impossibility. Tasks are additionally screened to exclude any instance that would reward unsafe, deceptive, or socially inappropriate behavior.

Third, diversification samples retained tasks to ensure coverage across objects, layouts, goals, relational structures, interaction lengths, and constraint combinations. Redundant instances are reduced; underrepresented configurations are supplemented. Difficulty bands, cross-context splits, and held-out generative combinations can be introduced to evaluate transfer and generalization directly rather than infer them from aggregate performance.

Lastly, evolution uses observed patterns of success and failure to generate new variants that preserve the target construct while modifying surface form, contextual constraints, or interaction dynamics. The purpose is not to produce arbitrarily harder tasks but to probe whether performance reflects transferable structure. Tasks that are consistently passed may be varied to identify the boundaries of generalization; tasks that produce inconsistent performance may be decomposed to isolate the source of variance.

To preserve reproducibility and fairness within this open-ended design, the evaluation procedure should specify construct definitions, parameter ranges, task seeds, sampling protocols, and contamination-control mechanisms in advance. For a given evaluation round, task instances can be generated from pre-registered components and random seeds, allowing the same sample to be reproduced for audit or cross-system comparison. When comparing multiple systems, the same seeded task set should be used. When evaluating the same system repeatedly over time, however, fixed-sample reassessment should be distinguished from new-sample generalization testing. Fixed samples are useful for estimating test–retest stability, whereas newly sampled but structurally equivalent task instances are needed to evaluate generalization beyond the original items. To reduce data contamination and task memorization, evaluation seeds, generated item pools, and held-out task variants should be protected before testing and disclosed only after evaluation. Repeated evaluations should therefore use rotated or newly generated held-out samples from the same construct space, with item-level overlap recorded and controlled.

A full GSA evaluation is necessarily more demanding than a fixed benchmark because it separates forms of evidence—generalization, value-oriented regulation, autonomy, specialized stability, and deployment reliability—that a single aggregate score cannot establish. This complexity is purposeful rather than mandatory at the same level for every study. GSA can be implemented modularly: a lightweight research evaluation may use a limited number of expert-defined task families, reproducible seeded generation, automated feasibility checks, and targeted human review of ambiguous cases. More intensive requirements, such as simulator maintenance, protected held-out task pools, adversarial testing, expert value scoring, and secure seed governance, become necessary when claims concern high-stakes deployment or certification-like assessment. Evaluators should report the modules implemented, the scope of the claims, and the associated computational and human-review costs so that the evaluation burden remains proportionate to its intended use.

Together, this generative mechanism gives G-stage evaluation its open-ended character. The task distribution is not closed at the point of evaluation design; instead, it can be expanded as new task families are defined and new generative variables are introduced. In this sense, the dynamic task-generation procedure operationalizes the G-stage requirement of open-ended task generalization: the system should be able to handle tasks drawn from a potentially expandable yet structurally coherent generative space, including task variants not explicitly included in the evaluation prompts or task-specific training procedure.

At the S stage, the same pipeline components apply, but the generative space is bounded by the target domain: task families are more narrowly defined, and the evaluation criteria shift from cross-family transfer to within-domain stability and repeatability.

At the A stage, the pipeline is instantiated in realistic deployment settings, where generative variables include environmental disturbances, underspecified user goals, and long-horizon interaction dynamics rather than controlled laboratory configurations. In all three cases, the underlying logic is the same: construct definition precedes task generation, generative variables are explicit and documented, and the resulting task distribution can be inspected and reproduced.

## 5. An Illustrative Example: Composite Household Tasks as G-Stage Evaluation Prototype

The preceding sections describe the G-stage requirements and the task-generation mechanism at a high level. This section presents an illustrative example of G-stage evaluation in practice. Rather than serving as a standalone AGI benchmark, this example illustrates how the GSA framework can be applied to a specific experimental setting and how identical performance data may lead to different interpretations under GSA compared with conventional benchmark approaches. This case study explores a different level of evaluation: interpreting model performance as stage-specific evidence within the GSA framework.

### 5.1. An Embodied Evaluation Approach of MLLM

The setting is a dynamic simulated home environment in which composite tasks are designed around everyday domestic activities. This context was selected because household activities provide a naturalistic setting in which intelligent agents must integrate contextual understanding, goal-directed reasoning, and adaptive decision-making rather than execute isolated skills. For example, preparing a table requires an agent to identify relevant objects, infer the intended context, organize items according to spatial and practical constraints, and determine whether the outcome satisfies the task goal. Similarly, packing luggage requires selecting goal-relevant items, excluding irrelevant objects, and adapting actions according to contextual requirements. The developmental inspiration lies in using familiar, ecologically meaningful activities that require the coordination of perception, object knowledge, spatial reasoning, language comprehension, action planning, and social appropriateness. Such tasks also require agents to transfer knowledge across contexts, align actions with contextual expectations, and organize goal-directed behavior under environmental constraints. In this prototype, the resulting performance provides preliminary evidence relevant to G-stage embodied competence; the three G-stage dimensions are not independently scored.

The task set includes eight types of embodied composite tasks: counting objects, building blocks, jigsaw puzzles, understanding buttons, setting tables, tidying up rooms, preparing baggage, and selecting gifts. These tasks were selected as representative examples of everyday activities that require models to integrate perception, reasoning, and action in context-rich environments. Table 5 summarizes how each task relates to the seven G-stage evaluation dimensions, illustrating the capability demands involved in different embodied scenarios.

For descriptive purposes, the tasks were organized into three broad categories according to their predominant characteristics. The object understanding category includes counting objects and selecting gifts, which emphasize object recognition, identification, and goal-relevant selection. The spatial intelligence category includes building blocks, jigsaw puzzles, and understanding buttons, which involve reasoning about spatial configurations, object relations, and functional interactions. The social activity category includes setting tables, tidying up rooms, and preparing baggage, which require agents to coordinate multiple subtasks under practical and socially situated goals. Notably, these categories serve as an organizational scheme for illustrating task characteristics rather than defining mutually exclusive cognitive constructs.

**Figure 2:**
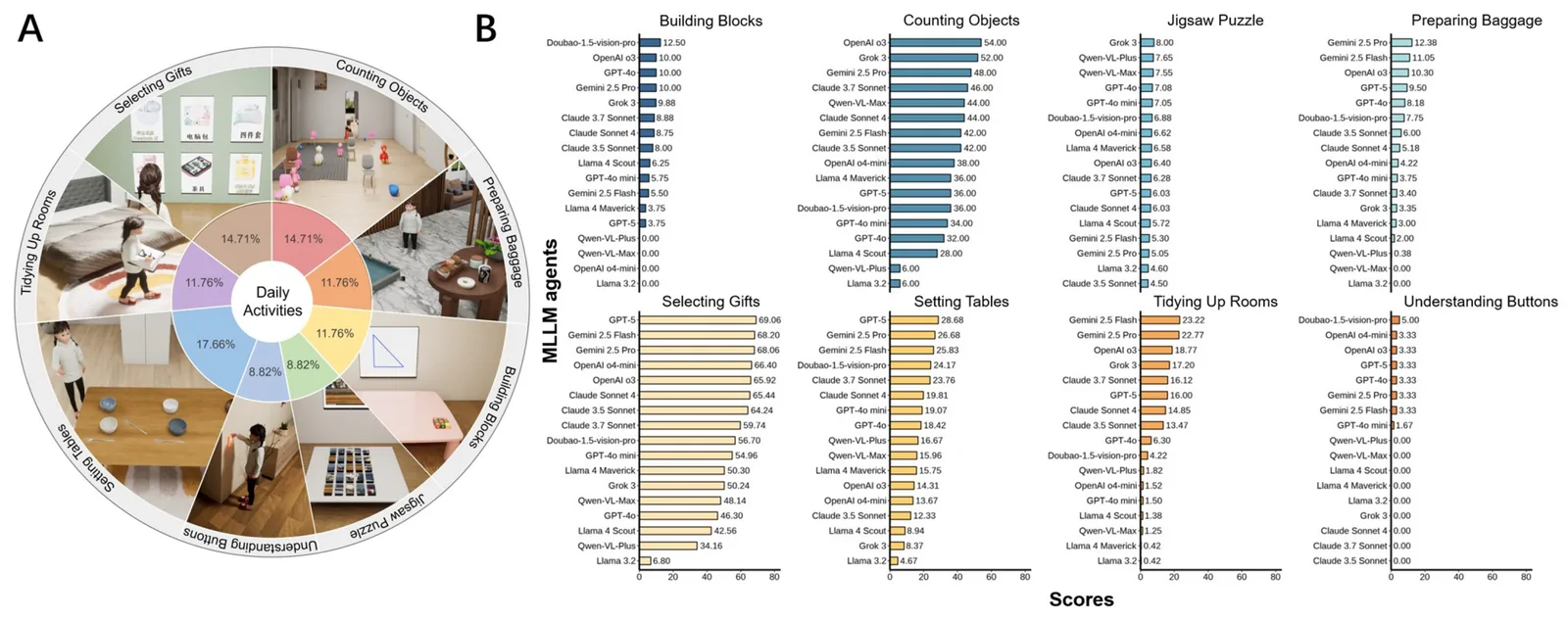
Developmentally inspired tests of embodied abilities. (**A**) Illustration of eight everyday household tasks used as ecologically grounded evaluation scenarios. (**B**) Performance of 17 leading proprietary and open-source MLLM agents across the eight tasks. The results show consistently limited performance across object understanding, spatial intelligence, and social activity tasks.

As shown in Figure 2, 17 representative proprietary and open-source multimodal large language models (MLLMs) were evaluated in a three-dimensional (3D) simulated household environment. In this environment, each MLLM-based agent controls a virtual human embodiment through standardized perception and action interfaces. The system initializes the simulated scene and controllable virtual human, and then provides a natural-language instruction, such as tidying a room or preparing a dining table. At each step, the agent receives multimodal observations from the environment, including visual views and structured object information. Based on these observations and the task goal, the agent generates an executable application programming interface (API) action, which is used to control the virtual human in the simulator. After each action, the environment returns an updated observation, enabling a closed perception–reasoning–action loop. The task terminates when the time limit is reached or when the agent issues a termination command.

Unlike conventional image–text question answering evaluations, this setting examines how MLLMs integrate perception, reasoning, and action in a sequential embodied environment. The resulting behavioral trajectories provide evidence relevant to how models handle changing observations, high-level goal decomposition, and action execution under environmental constraints.

### 5.2. MLLM Evaluation Results and Discussion

Results showed consistently limited performance across all three categories (Figure 2B), although performance varied across tasks and models. Overall, MLLM-based agents encountered substantial challenges in completing composite tasks within the simulated home environment. Even the best-performing model achieved only a modest average score, suggesting that current MLLMs still face difficulties in integrating multimodal information and sustaining reliable perception–reasoning–action processes in complex embodied settings.

A category-level breakdown showed that models generally performed relatively better in the object understanding category than in the spatial intelligence category and social activity category. In the object understanding category, models demonstrated relatively stronger performance on perceptual recognition tasks, such as counting objects, but performance declined when object information needed to be interpreted in relation to contextual goals. For example, selecting gifts required not only identifying candidate objects but also determining their relevance based on contextual requirements. These results suggest that current MLLMs can process object-level information to some extent but remain challenged when object knowledge must be flexibly integrated with situated contexts and task objectives.

Performance was generally weaker in the spatial intelligence category. Tasks such as building blocks, jigsaw puzzles, and understanding buttons required models to reason about spatial relations, configurations, and functional consequences of actions. The limited performance suggests that current MLLM-based agents face challenges in applying spatial knowledge across varying configurations and translating visual understanding into effective interactions with the environment. Although some models achieved relatively higher scores on individual spatial tasks, the absolute performance remained low, and no model demonstrated consistently reliable performance across the range of spatially demanding tasks included in this evaluation.

Performance in the social activity category also remained limited, although different models showed relative advantages across individual tasks. For example, some models performed better in setting tables, whereas others showed strengths in tidying rooms or preparing baggage. However, the leading scores remained modest. These tasks require agents to interpret high-level goals, organize multiple subtasks, and select actions consistent with practical and contextual expectations. The results indicate that current MLLMs still face challenges in sustaining autonomous and context-sensitive behavior in complex embodied environments.

To further examine whether the task battery reflected a single overall performance dimension or differentiated capability patterns, we analyzed inter-task correlations across the 17 model-level task scores (Appendix C). Task correlations were heterogeneous (r=−0.32 to 0.79, median = 0.47), with some tasks showing strong associations (e.g., counting objects and selecting gifts, r=0.79) and others exhibiting distinct performance profiles (e.g., jigsaw puzzles). This pattern suggests that the task battery captures heterogeneous capability demands and supports interpreting model performance through capability profiles rather than a single aggregate score.

From the perspective of G evaluation, these results are informative not because they produce a single ranking of models, but because they reveal how current MLLMs perform relative to different mechanisms emphasized in G-stage competence. The relatively stronger performance in the object understanding category suggests that current models show comparatively greater capability in processing object-level information and perceptual recognition. In contrast, weaker performance in the spatial intelligence category highlights challenges in spatial reasoning, functional understanding, and embodied planning. Limited performance in the social activity category further indicates difficulties in sustaining complex, context-sensitive, and goal-directed behavior. Thus, while a conventional benchmark interpretation may summarize these results as low overall performance, the GSA framework provides a more diagnostic perspective by identifying specific aspects of embodied intelligence that remain challenging for current models.

Importantly, this example represents one possible instantiation of G-stage evaluation rather than a complete implementation of the full GSA framework. The household setting was selected because it provides an ecologically grounded context involving interactions among perception, action, contextual understanding, and practical expectations. However, several aspects require further validation. First, future studies should establish stronger empirical validation between task characteristics and the intended G-stage mechanisms. Second, incorporating human or expert baselines would provide a more interpretable reference for positioning model performance relative to relevant baseline capabilities. Third, systematically designing tasks with calibrated difficulty levels would enable more fine-grained assessment of capability progression and failure patterns. In addition, the current evaluation was conducted in simulation rather than the physical world, and models were assessed through a constrained embodied interface. Future extensions should therefore incorporate broader environments, validated task structures, and more diverse embodied platforms. Because many task scores are concentrated near the lower end of the scale, possible floor effects and restricted variance may attenuate some inter-task correlations; the present correlation analysis should therefore be interpreted descriptively rather than as psychometric validation. Furthermore, a complete evaluation of the G-stage would require systematic and independent assessment of dimensions not fully captured by this prototype, particularly value-oriented regulation and autonomous task generation.

## 6. Discussion and Future Directions

This theoretical article has examined AGI evaluation through the lens of human intelligence research. It argues that benchmark-centered approaches, while valuable for comparing systems, provide limited insight into the cognitive–architectural organization, developmental progression, and real-world adaptive functioning that have long been central to intelligence research.

The GSA framework addresses this gap by organizing intelligence evidence into three progressive but non-substitutable levels. The G-stage concerns foundational abilities regarding cross-context generalization, autonomous task generation, and value-oriented regulation—assessed as integrated architectural properties rather than task-specific skills. The S-stage concerns the consolidation of these capacities into stable, repeatable domain competence under controlled conditions. The A-stage concerns whether such competence transfers to realistic environments involving uncertainty, long-horizon interaction, safety constraints, and the real costs of failure. The key contribution of the present GSA formulation is not simply to revisit a previously proposed developmental idea, nor to provide a replacement benchmark. It is to turn an initial developmental route into a stage-specific interpretation model linked to cognitive architecture: the same performance data yields different and more informative conclusions when read against the evidential requirements of each stage rather than as a single aggregate score.

Accordingly, the utility discussed here is framework-level rather than a completed empirical validation. GSA can turn benchmark results into stage-specific guidance: it distinguishes architectural readiness, domain competence, and deployment readiness; identifies whether weaknesses lie in generalization, value-oriented regulation, autonomy, robustness, or real-world reliability; and may help developers choose what to improve before applying AI in domains such as education, healthcare, robotics, scientific assistance, and public services. The illustrative case study illustrates this diagnostic use by reorganizing model scores into capability gaps, but it does not validate universal thresholds or stage boundaries. Future work should test GSA through multidimensional empirical tasks, human or expert baselines, longitudinal and cross-system validation, and studies of whether GSA-guided evaluation improves real AI development and deployment decisions.

Several limitations of the present framework should be acknowledged. First, the quantitative thresholds proposed in Section 3 (Equations (1)–(3) and the S-stage and A-stage criteria in Table 4) are illustrative rather than empirically calibrated. Their purpose is to show that stage assignment can be made explicit and auditable; the specific values require validation across diverse systems and domains before they can serve as normative standards. Second, although this revision provides a provisional scoring protocol for value-oriented regulation, measuring whether an agent possesses a stable internal value structure, rather than merely producing value-compliant outputs under observation, remains an open problem.

Third, stage boundaries may still prove ambiguous in practice: a system may satisfy some but not all criteria for a given stage, or may satisfy G-stage generalization requirements within one domain while failing them in another. The multidimensional profiling rule proposed above is a practical starting point, but it requires empirical calibration and community scrutiny. Fourth, a full GSA evaluation is more resource-intensive than a conventional fixed benchmark. This burden is partly inherent to separating forms of evidence that simpler benchmarks leave unmeasured, but it can be controlled through the modular implementation described in Section 4. A narrow benchmark may be sufficient for a narrow, low-risk claim, but it should not be interpreted as evidence for the full GSA profile. Fifth, the developmental framing of the household prototype is heuristic. The tasks are inspired by familiar activities that coordinate multiple capacities; they should not be interpreted as child-equivalent tests or as measures of human developmental milestones.

These limitations suggest several directions for future research. First, the proposed pipeline should be empirically instantiated across a wider range of domains beyond the household scenario illustrated in Section 5. Second, future studies should include multidimensional validation, including inter-task reliability, dimensionality analysis, human or expert baselines, and longitudinal test–retest designs. Third, the provisional scoring rules for value-oriented regulation, autonomy, and stage assignment should be calibrated across larger and more diverse samples of AI systems. Fourth, the generalizability of GSA should be examined in other domains, including embodied agents, educational AI, healthcare AI, scientific-assistance systems, and human-centered AI services. More broadly, the GSA framework aims to contribute to a shared vocabulary between AI evaluation and human intelligence research. Concepts such as construct validity, developmental staging, ecological validity, and adaptive functioning may be usefully extended to artificial systems, even when those systems do not reproduce human cognitive architecture. Establishing and empirically testing this conceptual bridge between the two fields remains an important long-term goal.   

## Figures and Tables

**Table 1:** Ability Requirements Across G, S, and A Evaluation Levels.

Dimension	G-Stage	S-Stage	A-Stage
Vision	Recognize basic objects and scenes (e.g., cats, tables, people)	Analyze complex visual attributes (e.g., emotions, textures, medical features); apply visual understanding for domain-specific decision-making (e.g., industrial inspection, traffic monitoring).	Perform goal-directed visual tasks in real-world scenarios (e.g., real-time quality inspection, medical image–guided operations).
Learning	Learn simple patterns from limited data (e.g., linear regression, basic classification).	Transfer and generalize learned knowledge to new tasks (e.g., few-shot learning, domain adaptation).	Continuously learn and self-optimize in open, dynamic environments (e.g., online learning, lifelong adaptation).
Cognition and Reasoning	Identify and understand basic logical relations (e.g., “If A, then B”).	Perform multi-step reasoning integrating multiple information sources (e.g., causal inference from short passages).	Conduct complex reasoning for real-world decision-making (e.g., medical diagnosis, legal reasoning, strategic planning).
Natural Language	Understand and generate basic sentences (e.g., question answering (QA), named entity recognition).	Understand complex semantic relations and perform multi-turn dialogue; deliver domain-specific professional language functions (e.g., counseling, academic writing, legal document generation).	Execute language tasks embedded in real-world workflows (e.g., clinical documentation assistance, enterprise communication).
Motor Control	Execute basic physical actions or simulations (e.g., grasping, simple obstacle avoidance).	Coordinate multi-step actions in dynamic environments (e.g., object transport, assembly).	Perform goal-directed physical tasks in complex real-world settings (e.g., robotic surgery, autonomous driving, warehouse logistics).
Value	Recognize basic value orientations (e.g., good/bad, safe/unsafe, social norms).	Conduct value trade-offs in moral dilemmas; generate ethically consistent recommendations based on social norms.	Apply value-consistent decision-making in high-impact real-world contexts (e.g., healthcare triage, public policy implementation, autonomous governance).

**Table 2:** Application-Level (A-Stage) Evaluation Scenarios Across Virtual and Real-World Domains.

Evaluation Aspect	Virtual Scenarios	Real-World Scenarios
Deployment Context	Digitally mediated service environments that involve long-horizon interaction with human users.	Physically grounded operational environments with embodiment constraints, sensor noise, and safety risks.
Representative Tasks	Legal consultation, music composition, domestic dialogue systems, and personal assistants.	Precision assembly, autonomous navigation, and emergency decision-making in high-stakes settings.
Deployment Abilities	Maintaining interaction coherence, tracking goals over time, and sustaining reliable service behavior across extended interactions.	Integrating perception and action, controlling motion, reasoning spatially, and adapting plans in real time under physical constraints
Value and Safety Considerations	Compliance-aware reasoning, emotional sensitivity, user trust, and socially appropriate behavior under underspecified user intent.	Risk-sensitive planning, safety compliance, and value-aligned action under physical constraints.
Failure Characteristics	Hallucination, inconsistency, service degradation, or loss of user trust.	Physical safety violations, execution failure, instability, or irreversible real-world consequences.

**Table 3:** Comparison Between Conventional Evaluation and the GSA Framework.

Dimension	Conventional Evaluation	GSA Framework
Goal	Assess performance on predefined tasks	Assess general ability in open-ended environments
Task Setting	Fixed tasks with explicit input–output specifications; examples include GLUE, MMLU, MATH, MMBench, HellaSwag, and TruthfulQA.	Open-world physical and social scenarios with partially unknown task structures; examples include dynamically generated household tasks, embodied interaction tasks, and open-ended virtual-agent scenarios.
Evaluation Mode	Static snapshot of current competence under predefined conditions.	Value-driven generalization and adaptive behavior over time through dynamic task generation, task diversification, and longitudinal evaluation.
System Role	Task-specific tool executing designated functions, such as question answering, text completion, image classification, or mathematical reasoning.	Autonomous agent adapting to novel tasks and environments, including goal generation, action planning, social interaction, and safety-sensitive decision-making.
Interpretation	Benchmark-level proficiency within a bounded task distribution.	Developmental potential, architectural readiness, robustness, transferability, and deployment-level applicability.

**Table 4:** Illustrative A-Stage Metrics and Example Operational Thresholds for Realistic Deployment.

Metric	Definition	Threshold
Task Completion Rate	Proportion of realistic use-case tasks completed successfully, including novel scenarios not encountered during S-stage evaluation	≥85%
Autonomy Ratio	Proportion of task attempts that succeed without human intervention	≥90%
Recovery Rate	Proportion of partial failures from which the system autonomously recovers and continues execution	≥70%
Critical Safety Incident Rate	Proportion of task attempts resulting in injury, property damage, or irreversible consequence	≤1%
Value Consistency Rate	Proportion of decisions that remain consistent with predefined value and safety constraints	≥90%

**Table 5:** Illustrative mapping between embodied composite household tasks and G-stage evaluation dimensions. Checkmarks indicate that a task involves the corresponding capability demand.

Dimension	Capability	Counting Objects	Building Blocks	Jigsaw Puzzles	Understanding Buttons	Setting Tables	Tidying Up Rooms	Preparing Baggage	Selecting Gifts
F1: Generalization	Physical Intelligence	✓	✓	✓	✓	✓	✓	✓	✓
	Social Intelligence					✓	✓	✓	✓
F2: Value-Oriented	Value Acquisition					✓	✓	✓	✓
	Value-driven Behavior					✓	✓	✓	✓
F3: Autonomy	Explainability	✓	✓	✓	✓	✓	✓	✓	✓
	Security and Trust				✓	✓		✓	
	Self-awareness		✓	✓	✓			✓	

**Figure A1:**
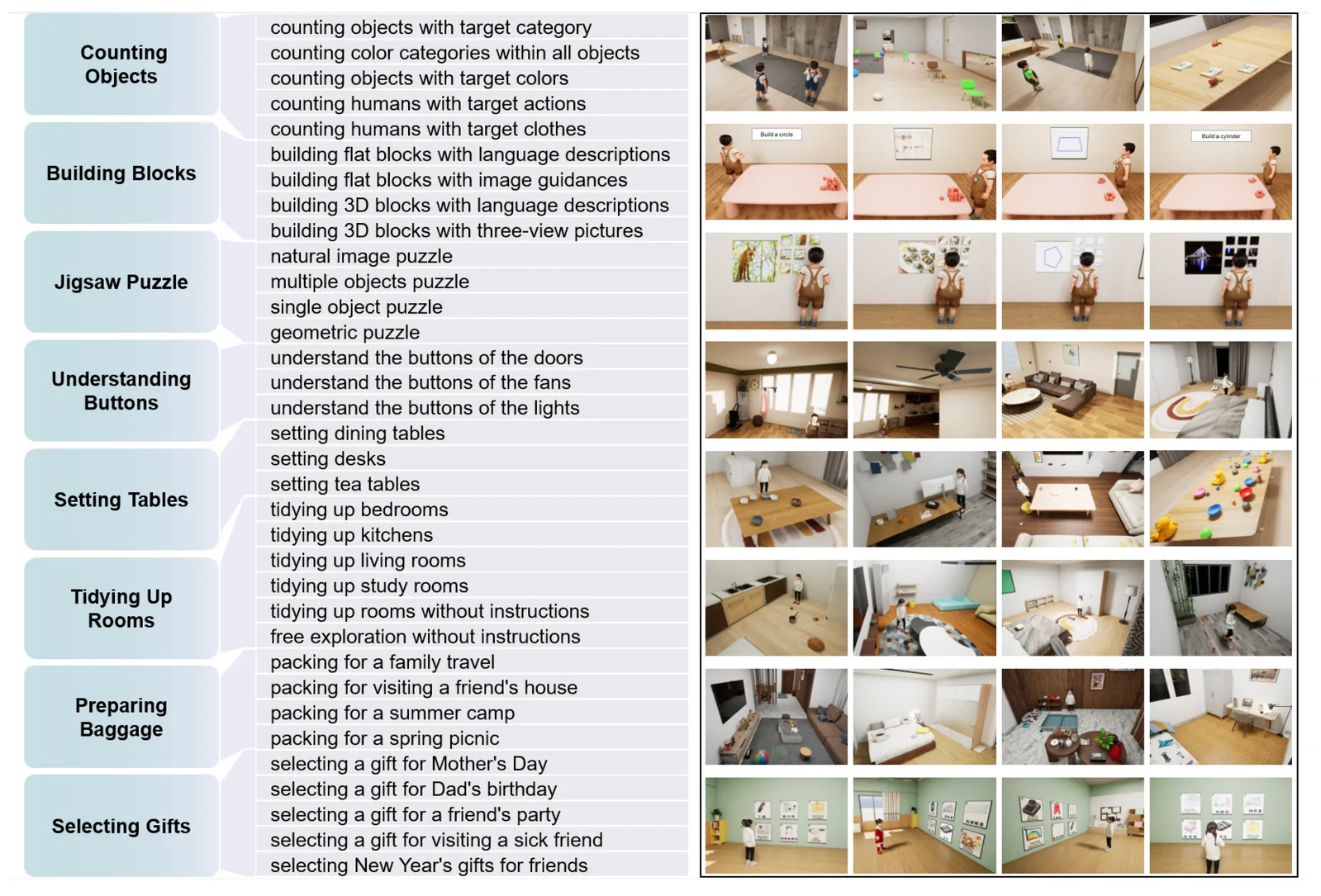
Overview of the eight task types with representative subtasks.

**Figure A2:**
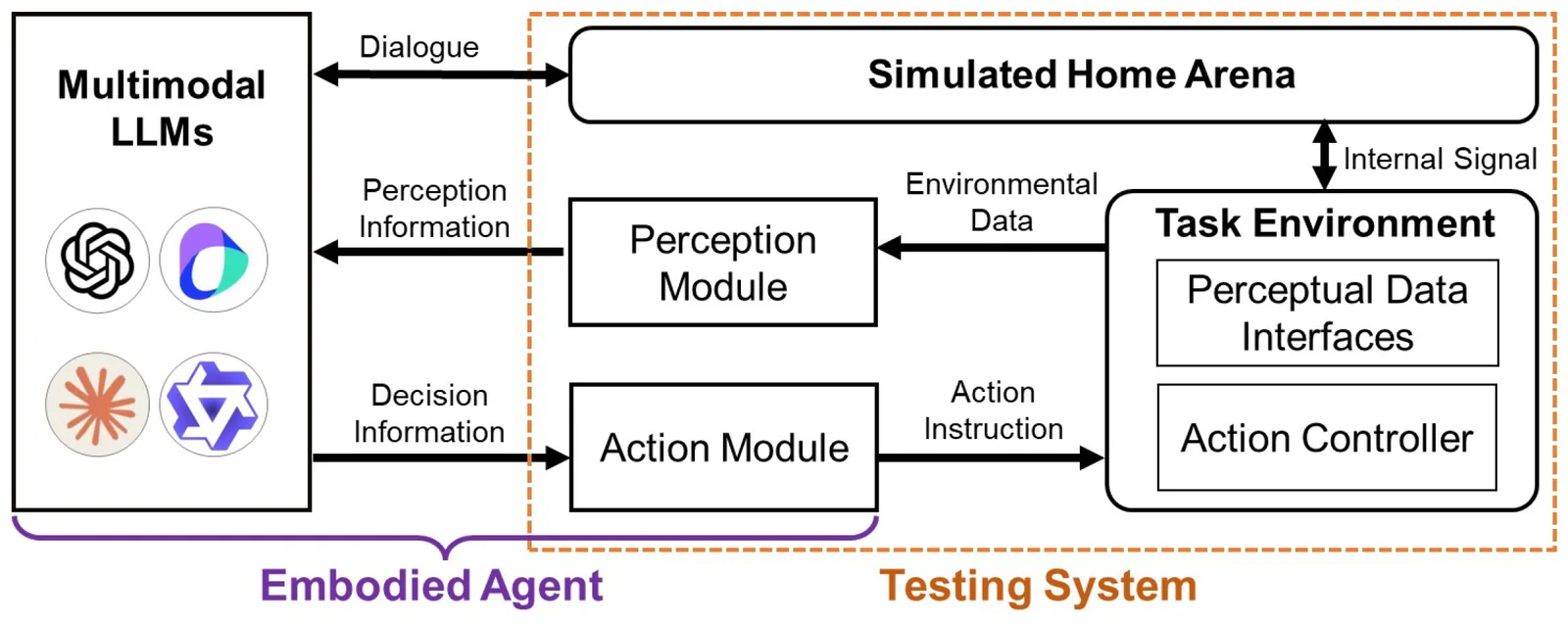
System framework for evaluating MLLM-based embodied agents.

**Figure A3:**
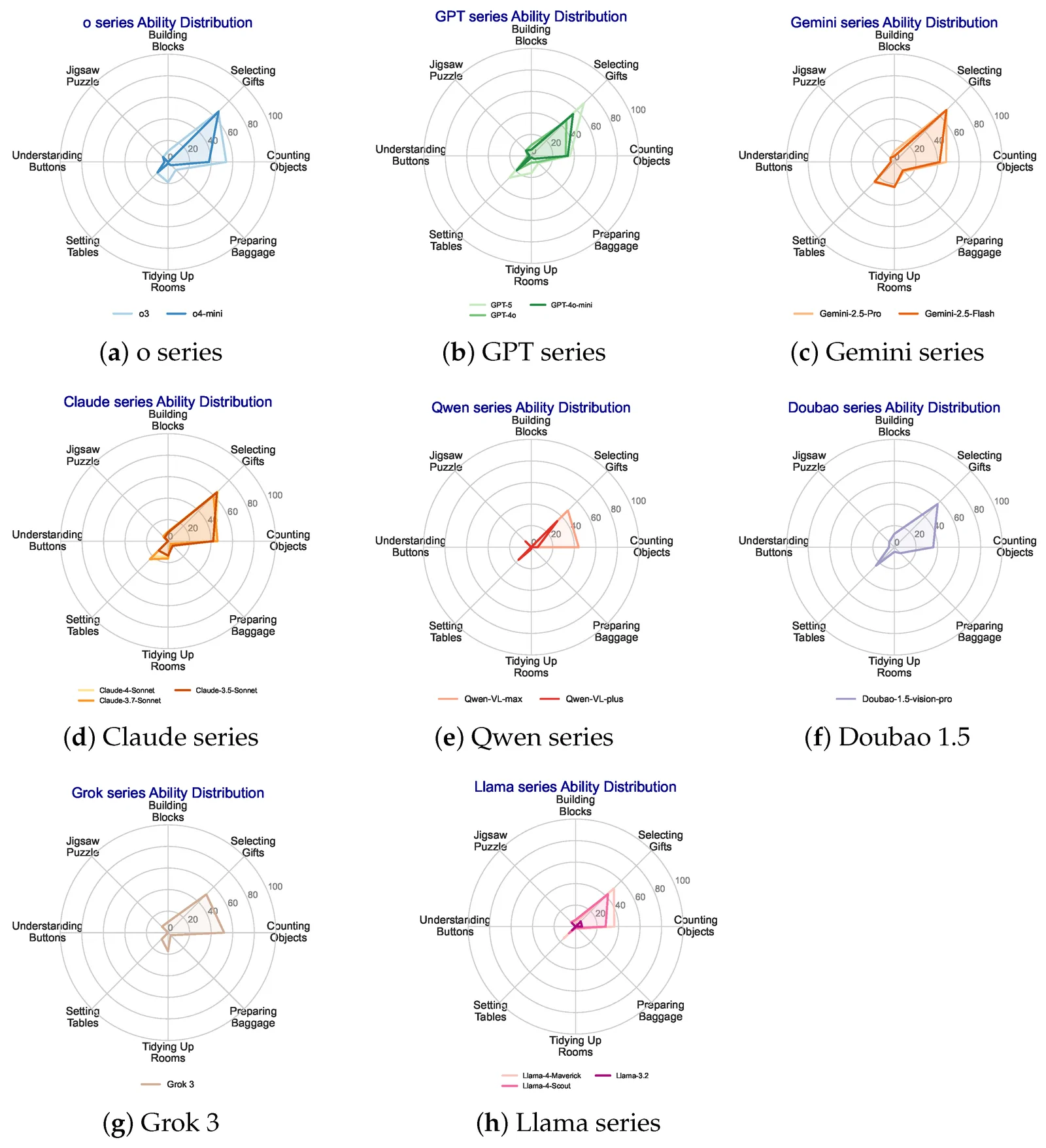
Ability distribution of 8 series of MLLM agents across eight task categories.

## Data Availability

The code, model outputs, and analysis records supporting the illustrative case study are not publicly available because the materials are subject to institutional and intellectual property restrictions. Requests for access may be directed to the corresponding authors and will be considered subject to the applicable restrictions.

## Abbreviations

The following abbreviations are used in this manuscript:

AGI	Artificial General Intelligence
AI	Artificial Intelligence
CUV	Cognitive architecture, potential functions, and value functions
GSA	General–Specialized–Applicable framework
IQ	Intelligence Quotient
MLLM	Multimodal Large Language Model
API	Application Programming Interface
ATG	Autonomous Task Generation
BIG-bench	Beyond the Imitation Game Benchmark
GLUE	General Language Understanding Evaluation
HELM	Holistic Evaluation of Language Models
JSON	JavaScript Object Notation
MMBench	Multi-modal Benchmark
MMLU	Massive Multitask Language Understanding
PARTNR	Planning And Reasoning Tasks in humaN–Robot collaboration
QA	Question Answering
ReAct	Reasoning and Acting
RGB	Red–Green–Blue
SuperGLUE	Super General Language Understanding Evaluation
TC	Task Coverage
TR	Task Relevance
VCR	Value Consistency Rate
3D	Three-Dimensional

## Appendix A. Details of Tasks

**Task 1: Counting Objects**

- **Task Description**: The agent was located in a room, and it was required to walk around the room and count the number of objects with specific attributes.
- **Input/Output**: Inputs included a task instruction and first-person view images; outputs were text-based responses.
- **Metric**: Response accuracy. No response equals failure.
- **Contents**: (1) counting objects with target category; (2) counting color categories within all objects; (3) counting objects with target colors; (4) counting humans with target actions; (5) counting humans with target clothes.

**Task 2: Building Blocks**

- **Task Description**: An agent is positioned in front of a table with a stack of small cubes. Its task is to assemble the blocks into a shape that matches a goal state defined by either a language instruction or a 2D image cue.
- **Input/Output**: Inputs included a task instruction, first-person view images, object information, and an action list; outputs were action sequences.
- **Metric**: The similarity between the final block state and the target stage, which is evaluated by an automatic scoring system or human experts.
- **Contents**: (1) building flat blocks with language descriptions; (2) building flat blocks with image guidance; (3) building 3D blocks with language descriptions; (4) building 3D blocks with three-view pictures.

**Task 3: Jigsaw Puzzle**

- **Task Description**: Given the target image, the agent was required to replicate the target image by manipulating the square blocks with different image patterns.
- **Input/Output**: Inputs included a task instruction, first-person view images, object information, and an action list; output was action sequences.
- **Metric**: Each block placed in the correct position contributes to the partial credit, as defined by the task environment.
- **Contents**: (1) natural-image puzzle; (2) multiple-object puzzle; (3) single-object puzzle; (4) geometric puzzle.

**Task 4: Understanding Buttons**

- **Task Description**: The agent was located in a room with many buttons that can be manipulated, and the agent was required to discover the function of each button through interaction.
- **Input/Output**: Inputs included a task instruction, first-person view images, object information, and an action list; outputs were text-based responses.
- **Metric**: Response accuracy. No response equals failure.
- **Contents**: (1) understand the buttons of the doors; (2) understand the buttons of the fans; (3) understand the buttons of the lights.

**Task 5: Setting Tables**

- **Task Description**: Given a cluttered desktop layout, the agent needs to organize it into a reasonable target state as required.
- **Input/Output**: Inputs included a task instruction, first-person view images, object information, and an action list; outputs were action sequences.
- **Metric**: The rationality and neatness of desktop object placement are evaluated by an automatic scoring system or human experts.
- **Contents**: (1) setting dining tables; (2) setting desks; (3) setting tea tables.

**Task 6: Tidying Up Rooms**

- **Task Description**: Given a cluttered room state, the intelligent agent needed to organize items according to the instructions.
- **Input/Output**: Inputs included a task instruction, first-person view images, object information, and an action list; outputs were action sequences.
- **Metric**: The proportion of correctly stored items to all items that should be processed.
- **Contents**: (1) tidying up bedrooms; (2) tidying up kitchens; (3) tidying up living rooms; (4) tidying up study rooms; (5) tidying up rooms without instructions; (6) free exploration without instructions.

**Task 7: Preparing Baggage**

- **Task Description**: The agent was required to find reasonable objects and pack them into the suitcase based on the provided contextual information.
- **Input/Output**: Inputs included a task instruction, first-person view images, object information, and an action list; the outputs were action sequences.
- **Metric**: Based on the task configuration, each item is assigned a different point score, and the final total score will be normalized to within 100 according to a pre-defined formula.
- **Contents**: (1) packing for a family trip; (2) packing for visiting a friend’s house; (3) packing for a summer camp; (4) packing for a spring picnic.

**Task 8: Selecting Gifts**

- **Task Description**: The agent is required to select gifts for given scenarios from a gift set.
- **Input/Output**: Inputs included a task instruction and first-person view images; outputs were text-based responses.
- **Metric**: Response accuracy. No response equals failure.
- **Contents**: (1) selecting a gift for Mother’s Day; (2) selecting a gift for Dad’s birthday; (3) selecting a gift for a friend’s party; (4) selecting a gift for visiting a sick friend; (5) selecting New Year’s gifts for friends.

## Appendix B. Technical Details of the Embodied MLLM Evaluation

In the MLLM-driven embodied agent framework, we established a perception–reasoning–action loop in a high-fidelity 3D simulator. The MLLM agents interacted with the environment by calling pre-defined function interfaces.

**Problem Definition.** At each timestep *t*, the MLLM agent receives an observation: $o_{t}=\left\{I_{t},\Sigma_{t}\right\}$, where $I_{t}=[I_{t}^{-45^{\circ }}\;∥\;I_{t}^{0^{\circ }}\;∥\;I_{t}^{+45^{\circ }}]$ denotes the concatenation of three red–green–blue (RGB) views (combining the left-front $45^{\circ }$, frontal, right-front $45^{\circ }$ views for a broader vision) and $\Sigma_{t}$ is a JavaScript Object Notation (JSON)-encoded scene description, with each object indexed by a unique object_id (e.g., object_desk_01) and annotated with name, color, position, and type. Given a natural-language goal *g*, the policy $\pi_{\theta }$ from the MLLM outputs a symbolic action $a_{t}$ (application programming interface (API) name and arguments), which is executed in the environment to produce $o_{t+1}$ until a termination condition is met.

**Framework Overview.** As shown in Figure A2, the framework comprises five elements: (1) *Perception*: acquire concatenated RGB triplets and object JSON from the testing environment; (2) *Semantic Packaging*: serialize $I_{t}$, $\Sigma_{t}$, and *g* into a structured prompt; (3) *Reasoning & Decision*: the MLLM generates a Reasoning and Acting (ReAct)-style reasoning trace $r_{t}$ and API call $a_{t}$; (4) *Execution*: dispatch atomic or macro actions to the simulator; (5) *Loop & Logging*: obtain next observation and record $\left(o_{t},r_{t},a_{t},o_{t+1}\right)$.

**Action Space & Tool Schema.** The action set A includes (1) *Atomic actions*: e.g., MoveForward(), PickUp(object) and (2) *Macro-actions*: e.g., MoveandPickUp(object), either executed directly or expanded into primitives. All APIs are defined in the system prompt as JSON entries specifying name, arguments, preconditions, and effects.

**ReAct-style Inference.** At each step, the policy outputs $\left(r_{t},a_{t}\right)$, where $r_{t}$ is a natural-language reasoning trace (non-executable) and $a_{t}$ is an executable API call. This design ensures interpretability while constraining actions to a safe, finite tool space.

**System Prompt Design.** For example, consider the task $g_{0}$ “You have three minutes to tidy up the dining table BP_DiningTable_06_C_0 to prepare it for three people”, an illustrative system prompt for this task is given below, where the testing system (called “Simulated Home Arena”) can recursively interact with the agent through language.

## Appendix C. Detailed Results of the Embodied MLLM Evaluation

This appendix reports additional result details for the embodied MLLM evaluation example discussed in Section 5.1.

**Overall Results.** Table A1 presents the evaluation results in eight embodied tasks, along with the average performance. Overall, MLLM agents showed limited performance on composite tasks grounded in home environments. The highest average score was achieved by Gemini-2.5-Pro, reaching only 24.53 out of 100. The proprietary models generally outperformed the open-source ones, although Qwen-VL-plus performed within the range of the open-source models, with a score of 8.33. Among the open-source models, the highest average score was 14.48 by Llama-4-Maverick. Nevertheless, the performance gaps between the proprietary and open-source models were not substantial. These results suggest that while the evaluated MLLMs showed comparatively stronger performance in perceptual recognition, their architectures, training regimes, and multimodal integration strategies may still be insufficient for achieving general-purpose embodied intelligence.

**Table A1:** Model Performance Comparison.

Model	Object Understanding		Spatial Intelligence			Social Activity			Mean
	Counting Objects	Selecting Gifts	Building Blocks	Jigsaw Puzzle	Understanding Buttons	Setting Tables	Tidying Up Rooms	Preparing Baggage	
Gemini-2.5-Pro	48.00	68.06	10.00	5.05	3.33	26.68	22.77	**12.38**	**24.53**
Gemini-2.5-Flash	42.00	68.20	5.50	5.30	3.33	25.83	**23.22**	11.05	23.05
o3	**54.00**	65.92	10.00	6.40	3.33	14.31	18.77	10.30	22.88
GPT-5	36.00	**69.06**	3.75	6.03	3.33	**28.68**	16.00	9.50	21.54
Claude-3.7-Sonnet	46.00	59.74	8.88	6.28	0.00	23.76	16.12	3.40	20.52
Claude-4-Sonnet	44.00	65.44	8.75	6.03	0.00	19.81	14.85	5.18	20.51
Doubao-1.5-vision-pro	36.00	56.70	**12.50**	6.88	**5.00**	24.17	4.22	7.75	19.15
Claude-3.5-Sonnet	42.00	64.24	8.00	4.50	0.00	12.33	13.47	6.00	18.82
Grok 3	52.00	50.24	9.88	**8.00**	0.00	8.37	17.20	3.35	18.63
o4-mini	38.00	66.40	0.00	6.62	3.33	13.67	1.52	4.23	16.72
GPT-4o	32.00	46.30	10.00	7.08	3.33	18.42	6.30	8.18	16.45
GPT-4o-mini	34.00	54.96	5.75	7.05	1.67	19.07	1.50	3.75	15.97
Qwen-VL-max	44.00	48.14	0.00	7.55	0.00	15.96	1.25	0.00	14.61
Llama-4-Maverick	36.00	50.30	3.75	6.58	0.00	15.75	0.42	3.00	14.48
Llama-4-Scout	28.00	42.56	6.25	5.72	0.00	8.94	1.38	2.00	11.86
Qwen-VL-plus	6.00	34.16	0.00	7.65	0.00	16.67	1.82	0.38	8.33
Llama-3.2	6.00	6.80	0.00	4.60	0.00	4.67	0.42	0.00	2.81

*Note:* Bold values indicate the highest score for each task and the highest overall mean score across the evaluated models.

**Fine-Grained Performance Across Domains.** Breaking down the results into the three core domains, most models demonstrated relatively stronger performance in object understanding compared to spatial intelligence and social activity. These results suggest that while current MLLM agents retain some strength in perceptual recognition tasks, they continue to struggle with more integrated, real-world embodied intelligence.

**Performance varied across tasks and models.** In the object understanding domain, o3 achieved the highest score for counting objects (54.00), while GPT-5 led in selecting gifts (69.06). In contrast, Llama-3.2 performed the worst in both subtasks and consistently showed low scores in seven of the eight tasks, except for the Jigsaw Puzzle. This underperformance likely stems from its limitations in precise object recognition, classification, and contextual understanding.

In the spatial intelligence domain, Doubao-1.5-vision-pro achieved the highest score in building blocks (12.50) and understanding buttons (5.00), while Grok 3 led in jigsaw puzzle (8.00). These findings indicate that spatial reasoning and manipulation remain particularly challenging for most MLLM embodied agents.

In the domain of social activity, different models achieved the highest scores in different tasks: GPT-5 in setting Tables (28.68), Gemini-2.5-Flash in tidying up rooms (23.22), and Gemini-2.5-Pro in preparing baggage (12.38). However, despite leading their respective tasks, these scores remain limited. These results suggest that the evaluated MLLMs faced difficulties with complex and goal-directed social understanding, and no evaluated model showed robust competence in this domain.
